# Risk of lymphoid malignancy associated with cancer predisposition genes

**DOI:** 10.1038/s41408-025-01283-z

**Published:** 2025-04-19

**Authors:** Nicholas J. Boddicker, Raphael Mwangi, Dennis P. Robinson, Cristine Allmer, Allison C. Rosenthal, Thomas M. Habermann, Andrew L. Feldman, Lisa M. Rimsza, Rebecca L. King, Melissa C. Larson, Bri J. Negaard, Aaron D. Norman, Nikhil Rajkumar, Stephen M. Ansell, Angela Dispenzieri, David L. Murray, Vincent Rajkumar, Shaji Kumar, Jithma P. Abeykoon, Regeneron Genetics Center, GNoncalo Abecasis, Aris Baras, Michael Cantor, Giovanni Coppola, Andrew Deubler, Aris Economides, Luca A. Lotta, John D. Overton, Jeffrey G. Reid, Katherine Siminovitch, Alan Shuldiner, John D. Overton, Christina Beechert, Caitlin Forsythe, Erin D. Fuller, Zhenhua Gu, Michael Lattari, Alexander Lopez, Maria Sotiropoulos Padilla, Manasi Pradhan, Kia Manoochehri, Thomas D. Schleicher, Louis Widom, Sarah E. Wolf, Ricardo H. Ulloa, Michael Cantor, Amelia Averitt, Nilanjana Banerjee, Dadong Li, Sameer Malhotra, Ob Deepika Sharma, Jeffrey Staples, Jeffrey G. Reid, Xiaodong Bai, Suganthi Balasubramanian, Suying Bao, Boris Boutkov, Siying Chen, Gisu Eom, Lukas Habegger, Alicia Hawes, Shareef Khalid, Olga Krasheninina, Rouel Lanche, Adam J. Mansfield, Evan K. Maxwell, George Mitra, Mona Nafde, Sean O’Keeffe, Max Orelus, Razvan Panea, Tommy Polanco, Ayesha Rasool, William Salerno, Jeffrey C. Staples, Kathie Sun, Goncalo Abecasis, Joshua Backman, Amy Damask, Lee Dobbyn, Manuel Allen Revez Ferreira, Arkopravo Ghosh, Christopher Gillies, Lauren Gurski, Eric Jorgenson, Hyun Min Kang, Michael Kessler, Jack Kosmicki, Alexander Li, Nan Lin, Daren Liu, Adam Locke, Jonathan Marchini, Anthony Marcketta, Joelle Mbatchou, Arden Moscati, Charles Paulding, Carlo Sidore, Eli Stahl, Kyoko Watanabe, Bin Ye, Blair Zhang, Andrey Ziyatdinov, Giovanni Coppola, Luca A. Lotta, Katherine Siminovitch, Alan Shuldiner, Bin Ye, Ariane Ayer, Aysegul Guvenek, George Hindy, Jan Freudenberg, Jonas Bovijn, Kavita Praveen, Manav Kapoor, Mary Haas, Moeen Riaz, Niek Verweij, Olukayode Sosina, Parsa Akbari, Priyanka Nakka, Sahar Gelfman, Sujit Gokhale, Tanima De, Veera Rajagopal, Gannie Tzoneva, Juan Rodriguez-Flores, Esteban Chen, Marcus B. Jones, Michelle G. LeBlanc, Jason Mighty, Lyndon J. Mitnaul, Nirupama Nishtala, Nadia Rana, Jaimee Hernandez, Grzegorz S. Nowakowski, Thomas E. Witzig, Anne J. Novak, Susan L. Slager, Celine M. Vachon, James R. Cerhan

**Affiliations:** 1 Division of Computational Biology, Mayo Clinic, Rochester, MN USA; 2 Division of Clinical Trials and Biostatistics, Mayo Clinic, Rochester, MN USA; 3 Department of Hematology/Oncology, Mayo Clinic, Phoenix, AZ USA; 4 Division of Hematology, Mayo Clinic, Rochester, MN USA; 5 Division of Hematopathology, Mayo Clinic, Rochester, MN USA; 6 Department of Laboratory Medicine and Pathology, Mayo Clinic, Rochester, MN USA; 7 Division of Hematopathology, Mayo Clinic, Phoenix, AZ USA; 8 Division of Epidemiology, Mayo Clinic, Rochester, MN USA; 9 University of Minnesota, Minneapolis, MN USA; 10 Regeneron Genetics Center, Tarrytown, NY USA

**Keywords:** Risk factors, Lymphoma, Myeloma

## Abstract

We investigated the prevalence of rare inherited pathogenic variants (PV) in 19 cancer predisposition genes regularly included on multi-gene panel testing based on NCCN guidelines and their association with the risk of lymphoid malignancies (LM) overall and by common lymphoma subtypes and multiple myeloma. The study population included newly diagnosed LM cases (*N* = 6990) and unrelated controls (*N* = 42,632), excluding individuals with a history of hematologic malignancy. Whole exome sequencing was performed on DNA from whole blood. PV were defined as loss-of-function (i.e., nonsense, frameshift, consensus splice sites) or identified as “pathogenic” or “likely pathogenic” in the ClinVar database. A total of 1816 (3.7%) individuals had a PV across the 19 genes, higher in cases (4.7%) than controls (3.5%). In controls, *CHEK2* (1.0%), *ATM* (0.4%), *BRCA2* (0.4%), and *BRCA1* (0.3%) had the highest prevalence. *ATM* (odds ratio [OR] = 1.86, 95% confidence interval [CI]: 1.36–2.49), *CHEK2* (OR = 1.74, 95% CI: 1.42–2.13) and *TP53* (OR = 9.07, 95% CI: 4.51–18.87) were associated with increased risk of LM overall and were further validated in the UK Biobank. We observed heterogeneity in associations by LM subtype. These results demonstrate that several commonly tested cancer predisposition genes are associated with an increased risk of LM.

## Introduction

Lymphoid malignancies (LM) are neoplasms that arise from the lymph nodes, other lymphatic tissue, or in the lymphatic cells of other organs. They are heterogenous with respect to biology, aggressiveness, and treatment, with over 100 subtypes including the common subtypes of diffuse large B-cell lymphoma (DLBCL), follicular lymphoma (FL), mantle cell lymphoma (MCL), marginal zone lymphoma (MZL), small lymphocytic leukemia (SLL), T-cell lymphoma (TCL), Hodgkin lymphoma (HL), Waldenström macroglobulinemia (WM), and multiple myeloma (MM) [1, 2]. Collectively, there were an estimated 125,110 new cases in the United States in 2023 [3].

Established non-genetic risk factors for LM, or specific subtypes, include family history of hematologic cancer, infectious agents, immune dysregulation, and chemical/toxin exposure [4]. Genetic epidemiology studies of LM risk have shown that LM clusters in families and that family history, irrespective of subtype, is a risk factor for LM [5]. However, a family history of a specific subtype is generally most strongly associated with an increased risk of the same subtype [6]. In a large study that investigated over 150,000 individuals with hematological malignancies, family history of a specific subtype with risk of the same subtype was >4-fold for chronic lymphocytic leukemia, MCL, and HL and between 2 to 3-fold higher for DLBCL, FL, and MM [7]. This study also showed that a family history of a specific LM subtype is associated with a risk of other subtypes.

A family history of LM supports that inherited genetics play an important a role in the etiology of LM. Genome-wide association studies (GWAS) have identified susceptibility variants for risk of HL, CLL, FL, DLBCL, MZL, WM, and MM [8–12]. These variants, in the form of single nucleotide polymorphisms (SNPs), are relatively common with small effects on risk. However, the genetic component attributed to rare mutations with a risk of LM is less understood, particularly in the general population.

Genetic variants that alter the function of a gene, most notably protein-truncating, can increase the risk of disease. These variants, called pathogenic variants (PV), are rare in the general population. Some of the more common genes that harbor PV associated with cancer risk are genes associated with DNA damage repair. For example, PV in *BRCA1* and *BRCA2* are notably associated with increased risk of breast (5-10-fold increase) [13, 14] and ovarian (5-11-fold increase) cancer [15]. *CHEK2* is associated with breast cancer (~2-fold increase) [13, 14], prostate cancer (~4-fold increase) [16, 17], and thyroid cancer (1.6-3-fold increase) [18, 19]. *ATM* is associated with breast cancer (~2-fold)[13, 14], ovarian cancer (1.7-fold increase) [15], and pancreatic cancer (4-fold increase) [20]. Given the strong associations of PV in these genes with several solid tumor malignancies, individuals diagnosed with these cancers often qualify for genetic testing based on patient or clinical characteristics at the time of diagnosis (e.g., age at diagnosis, cancer subtype, or family history) through National Comprehensive Cancer Network (NCCN) criteria due to the increased likelihood they carry a PV in a known cancer predisposition gene [21]. Knowing mutation status can inform therapy for the individual and risk assessment and management for family members without cancer.

To date, our understanding of the relationship between PV in known cancer predisposition genes and risk of LM is limited, but some suggestive evidence of an association has come from small population studies, family studies, or predisposition syndromes. Increased incidence of lymphoma has been reported in Li Fraumeni syndrome (*TP53*) [22] and Lynch Syndrome (*MSH2, MLH1, MSH6*) [23, 24]. Survivors of pediatric or adolescent lymphomas (*N* = 1380) were enriched for *BRCA2* mutations compared to gnomAD [25]. A recent study from the Japanese Biobank found that PV in *BRCA1, BRCA2, ATM*, and *TP53* were associated with the risk of lymphoma overall [26]. However, further research with large sample sizes is necessary to understand the prevalence of PV and the risk of LM and within LM subtypes. Herein, we investigate the association between PV in 19 cancer predisposition genes commonly found on genetic testing panels and the risk of LM and major LM subtypes using a large clinic-based case-control study.

## Methods

### Study participants

#### Lymphoma cases

Lymphoma cases (excluding CLL and MM) included newly diagnosed Hodgkin and non-Hodgkin lymphomas from the Mayo component of the Molecular Epidemiology Resource (MER), a prospective observational cohort study of newly diagnosed lymphoma patients from the Iowa/Mayo Lymphoma Specialized Program of Research Excellence (SPORE) [27]. Eligibility for this analysis included age 18 years or older, enrolled from 2002 to 2018, and had an available blood sample for DNA sequencing. All pathology was centrally reviewed and classified according to the WHO Classification [28]. Participants were offered an enrollment questionnaire (completion rate 82%) and a risk factor questionnaire (completion rate 76%), which included family history of hematological malignancies. MM cases were recruited from individuals having a bone marrow biopsy as part of their clinical workup for MM diagnosis at Mayo Clinic. Eligibility for this analysis included MM cases ages 18 and older, seen between 1998 and 2022, who also had an available blood sample as a source of DNA for sequencing. All MM diagnoses were confirmed by a hematopathologist. Family history of hematologic malignancy was obtained through medical record abstraction or a risk factor questionnaire.

#### Controls

Controls were from the Mayo Clinic Biobank, which is a large-scale bio-repository of Mayo patients aged 18 years or older who were enrolled between 2009 and 2016, mainly from primary care clinics [29]. Participants completed an enrollment questionnaire, which included a personal and family history of hematological cancers. Participants provided a blood sample for DNA sequencing. Participants with a personal history of a hematologic malignancy were excluded from this analysis.

### DNA sequencing and bioinformatics analysis

Germline DNA samples were sequenced for exomes by Regeneron Genetics Center (RGS) using a high-throughput automated approach. The targeted bases were captured using the Twist Comprehensive Exome design. The captured libraries were sequenced on the Illumina NovaSeq 6000 system on S4 flow cells using paired-end 75 bp reads. Genetic variants were called using a Parabricks accelerated version of DeepVariant v0.10. High-quality sequence data (>20× read depth) was observed in >95% of samples. Variants were annotated and classified using the Biological Reference Repository, a toolkit for annotating variants using public and user-specific annotation resources in indexed JSON-encoded flat files (catalogs) [30]. PVs, defined as loss-of-function (i.e., nonsense, frameshift, consensus splice sites (±1 or 2)) or identified as “pathogenic” or “likely pathogenic” in the ClinVar database, were identified in 19 cancer predisposition genes (*ATM, BARD1, BRCA1, BRCA2, BRIP1, CDKN2A, CHEK2, MLH1, MRE11, MSH2, MSH6, NBN, NF1, PALB2, PTEN, RAD50, RAD51C, RAD51D, TP53*). PV in *TP53* were restricted to alternate allele fractions (calculated as the fraction between alternate allele reads and the total number of reads at a specific genomic position) between 0.3 and 0.7 in an attempt to exclude potential clonal hematopoiesis [31]. The distributions of the variant allele fraction for the PVs for each gene are in Fig. S1.

### United Kingdom Biobank (UKB)

To validate our findings, we utilized lymphoma patients and controls from the UKB. The UKB cohort is a prospective cohort of >500,000 individuals aged 40–70 years at the time of recruitment, of which 450,000 had whole exome sequencing (WES) [32]. We used the International Classification of Disease (ICD) codes to identify lymphoid (ICD code C80) or MM (C90) patients. Prevalent and incident cases were included in the analyses. Controls were defined as those with no personal history of any cancer. In total, 4772 cases and 389,731 controls were included. PVs were identified using the same definition as above. The analyses were performed under the UKB application no. 79864.

### Statistical analysis

Frequencies of PV in each gene were tabulated for cases and controls. The association between PV in each gene and overall LM risk were estimated using odds ratios (OR) and 95% confidence intervals (CI) from logistic regression models, adjusted for age (at the time of diagnosis for cases and at the time of enrollment into the biobank for controls) and sex. Separate analyses by LM subtype were also conducted. Further analyses were conducted stratified by age, sex, and family history of hematologic malignancy. Case-only analyses were performed to compare patient characteristics between PV carriers and non-carriers in genes significantly associated with the risk of LM. All analyses were performed in R (version 4.2.2). Statistical significance was set at *p* < 0.003 to account for multiple testing (0.05/19 genes) for the analysis of LM overall. For UKB, Fisher’s Exact test was used to compare the frequency of PV carriers between cases and controls by gene. Meta-analyses were performed across the Mayo Clinic (discovery) and UKB (validation) datasets for genes where the Mayo Clinic cases had at least 4 PV carriers. Meta-analyses were conducted using the ‘metafor’ package to fit random-effects models via the restricted maximum likelihood method.

## Results

### Patient characteristics

The Mayo Clinic study included a total of 6990 LM cases and 42,632 controls, all unrelated individuals (Table 1). The median age for cases and controls was 63 years (range 19–99 years). The proportion of males was 58.5% in cases and 41.7% in controls. In both cases and controls, EUR ancestry accounted for 96.5% of the study population, followed by 1.4% AFR ancestry. Self-reported positive family history of lymphoma or leukemia was 14.9% in cases and 8.6% in controls. As expected, increasing age (OR = 1.007 [continuous], 95% CI: 1.005–1.009), male sex (OR = 1.88, 95% CI: 1.76–2.00), and positive family history of first degree relative with a hematologic malignancy (OR = 1.92, 95% CI: 1.75–2.10) were all significantly associated with LM. Non-Hodgkin lymphoma subtypes (NHL, *n* = 4395), HL (*n* = 457), and MM accounted for 62.9%, 6.5%, and 30.6% of cases, respectively. Within NHL, the most prevalent subtype DLBCL (*n* = 1146, 26.1%), followed by FL (*n* = 1132, 25.8%), MZL (*n* = 444, 10.1%), TCL (*n* = 369, 8.4%), MCL (*n* = 325, 7.4%), SLL (*n* = 192, 4.4%), and other B-cell lymphomas (*n* = 787, 17.9%).

**Table 1:** Patient characteristics by case/control status and by lymphoid malignancy subtype.

	Overall		Subtype								
	Case (*N* = 6990)	Control (*N* = 42,632)	SLL (*N* = 192)	DLBCL (*N* = 1146)	FL (*N* = 1132)	HL (*N* = 457)	MCL (*N* = 325)	MM (*N* = 2138)	MZL (*N* = 444)	Other B-cell (*N* = 787)	T-Cell (*N* = 369)
Age											
*N*	6986	42,456	192	1146	1132	457	325	2134	444	787	369
Median	63	63	67	64	61	40	64	63	63	64	61
Range	18.00–94.00	18.00–99.00	41.00–86.00	18.00–94.00	19.00–92.00	18.00–89.00	31.00–94.00	25.00–92.00	18.00–91.00	19.00–94.00	18.00–88.00
Age category, *N* (%)											
Missing	4	176	0	0	0	0	0	4	0	0	0
<60	2804 (40.1%)	17,804 (41.9%)	49 (25.5%)	418 (36.5%)	517 (45.7%)	337 (73.7%)	100 (30.8%)	752 (35.2%)	179 (40.3%)	281 (35.7%)	171 (46.3%)
60+	4182 (59.9%)	24,652 (58.1%)	143 (74.5%)	728 (63.5%)	615 (54.3%)	120 (26.3%)	225 (69.2%)	1382 (64.8%)	265 (59.7%)	506 (64.3%)	198 (53.7%)
Sex, *N* (%)											
Missing	3	0	0	0	0	0	0	3	0	0	0
Female	2897 (41.5%)	24,870 (58.3%)	80 (41.7%)	483 (42.1%)	518 (45.8%)	211 (46.2%)	77 (23.7%)	834 (39.1%)	251 (56.5%)	299 (38.0%)	144 (39.0%)
Male	4090 (58.5%)	17,762 (41.7%)	112 (58.3%)	663 (57.9%)	614 (54.2%)	246 (53.8%)	248 (76.3%)	1301 (60.9%)	193 (43.5%)	488 (62.0%)	225 (61.0%)
Genetic ancestry, *N* (%)											
Missing	18	168	0	1	5	0	0	5	2	3	2
AFR	98 (1.4%)	608 (1.4%)	1 (0.5%)	4 (0.3%)	8 (0.7%)	7 (1.5%)	2 (0.6%)	57 (2.7%)	5 (1.1%)	6 (0.8%)	8 (2.2%)
AMR	79 (1.1%)	385 (0.9%)	0 (0.0%)	9 (0.8%)	13 (1.2%)	6 (1.3%)	4 (1.2%)	25 (1.2%)	12 (2.7%)	3 (0.4%)	7 (1.9%)
EAS	40 (0.6%)	318 (0.7%)	0 (0.0%)	5 (0.4%)	6 (0.5%)	3 (0.7%)	1 (0.3%)	12 (0.6%)	2 (0.5%)	6 (0.8%)	5 (1.4%)
EUR	6730 (96.5%)	40,961 (96.5%)	190 (99.0%)	1122 (98.0%)	1096 (97.2%)	438 (95.8%)	318 (97.8%)	2031 (95.2%)	422 (95.5%)	768 (98.0%)	345 (94.0%)
SAS	25 (0.4%)	192 (0.5%)	1 (0.5%)	5 (0.4%)	4 (0.4%)	3 (0.7%)	0 (0.0%)	8 (0.4%)	1 (0.2%)	1 (0.1%)	2 (0.5%)
Family history of hematologic malignancy, *N* (%)											
Missing	2228	3402	17	364	284	159	107	831	97	250	119
No	4053 (85.1%)	35,854 (91.4%)	153 (87.4%)	675 (86.3%)	719 (84.8%)	266 (89.3%)	188 (86.2%)	1093 (83.6%)	298 (85.9%)	444 (82.7%)	217 (86.8%)
Yes	709 (14.9%)	3376 (8.6%)	22 (12.6%)	107 (13.7%)	129 (15.2%)	32 (10.7%)	30 (13.8%)	214 (16.4%)	49 (14.1%)	93 (17.3%)	33 (13.2%)

Family history (first degree) of hematologic malignancy is self-reported.

*SLL* small lymphocytic lymphoma, *DLBCL* diffuse large B-cell lymphoma, *FL* follicular lymphoma, *HL* Hodgkin’s lymphoma, *MCL* mantle cell lymphoma, *MM* multiple myeloma, *MZL* marginal zone lymphoma, other B-cell lymphoma (includes Incomplete, Other NHL).

Genetic ancestry: AFR African, AMR admixed population, EAS East Asian, EUR European, SAS South Asian.

### Association of predisposition genes and lymphoid malignancies overall

**Fig. 1:**
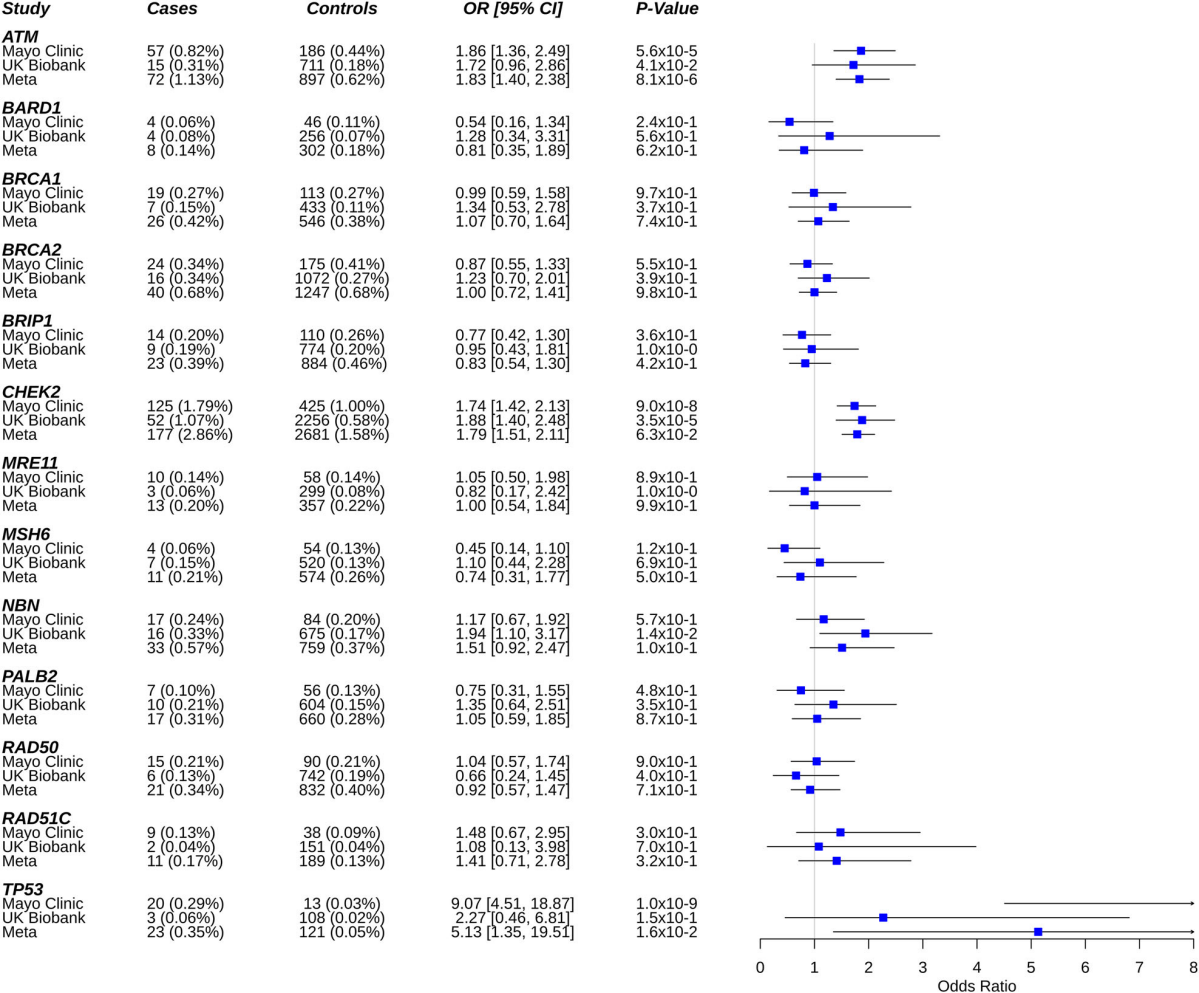
Association between pathogenic variants in cancer predisposition genes and risk of lymphoid malignancy. Estimates were adjusted for age and sex in the Mayo Clinic study. Univariate analysis was used in the UK Biobank cohort. OR Odds Ratio, CI Confidence Interval.

The prevalence of PV across the 19 cancer predisposition genes was 4.7% in cases and 3.5% in controls. Among cases, the highest prevalence of PV was observed for *CHEK2* (1.8%), *ATM* (0.8%), *BRCA1* (0.3%), *BRCA2* (0.3%), and *TP53* (0.3%, Fig. 1, Table S1). PVs in *ATM* (OR = 1.86, 95% CI: 1.36–2.49), *CHEK2* (OR = 1.74, 95% CI: 1.42–2.13), and *TP53* (OR = 9.07, 95% CI: 4.51–18.87) were associated with increased risk of LM (all *p* < 5.7 × 10^−5^). The remaining genes showed either no evidence of an association with the risk of LM (e.g., *BRCA1, BRCA2*) or had limited numbers of patients with PVs to assess associations (e.g., *CDKN2A, MLH1, MSH2, NF1, PTEN*, and *RAD51D*). The results remained unchanged when restricted to cases and controls who did not have a prior history of any cancer (excluding non-melanoma skin cancer, Table S2).

In the UKB cohort, we evaluated the 13 genes that had at least 4 cases with PV in the Mayo study. In the UKB cohort, the median age of cases was 65 years and 56 years in controls. The frequency of males was 55% for cases and 45% for controls.

We found significant associations with LM risk for both *ATM* (OR = 1.72, 95% CI: 0.96–2.86) and *CHEK2* (OR = 1.88, 95% CI: 1.40–2.48), with similar risk estimates as that observed in the Mayo study (Fig. 1). We also observed significant association of *NBN* (OR = 1.94, 95% CI: 1.10–3.17), which was not significant in the Mayo study. The association of LM risk with *TP53* was weaker (OR = 2.27, 95% CI: 0.46–6.81) than that in the Mayo study and not statistically significant in the UKB cohort. Similar to the Mayo cohort, no evidence of association was observed in the other genes. In a meta-analysis across the two cohorts (11,762 LM cases and 432,363 controls), we observed significant associations between risk of LM and *ATM* (OR = 1.83, 95% CI: 1.40–2.38), *CHEK2* (OR = 1.79, 95% CI: 1.51–2.11), and *TP53* (OR = 5.13, 95% CI: 1.35–19.51) (Fig. 1).

In the Mayo study, we performed stratified analyses for age (<60 years of age or ≥60 years of age), sex (male or female), and family history of hematologic malignancy (positive or negative first degree family history). Among participants under the age of 60 years (*n* = 2804 cases, *n* = 17,804 controls), the frequency of PVs was 4.9% in cases and 3.8% in controls. PVs in *CHEK2* (OR = 1.71, 95% CI: 1.24–2.32) and *TP53* (OR = 23.12, 95% CI:5.78–154.42) were associated with increased risk of LM in participants under age 60. Interestingly, *RAD51C* had an OR > 2 but did not reach statistical significance (*p* = 0.094, Table S3). Among those aged 60 years or older (*n* cases = 4182, *n* controls = 24,652), the frequency of PVs was 4.5% in cases and 3.2% in controls. PVs in *ATM* (OR = 2.10, 95% CI: 1.42–3.04), *CHEK2* (OR = 1.74, 95% CI: 1.32–2.26), and *TP53* (OR = 6.03, 95% CI: 2.57–14.17) were all significantly associated with the risk of LM. Stratifying by sex, among males (*n* cases = 4090, *n* controls = 17,762), the frequency of PV was 4.5% in cases and 3.7% in controls (Table S4). PVs in *ATM* (OR = 2.04, 95% CI: 1.36–3.00), *CHEK2* (OR = 1.33, 95% CI: 1.00–1.74), and *TP53* (OR = 12.52, 95% CI: 4.77–38.83) were significantly associated with the risk of LM. For females (*n* cases = 2897, *n* controls = 24,870), the frequency of PVs was 4.9% in cases and 3.4% in controls. Similarly, PVs in *ATM* (OR = 1.64, 95% CI: 0.99–2.59), *CHEK2* (OR = 2.45, 95% CI: 1.82–3.26), and *TP53* (OR = 6.09, 95% CI: 2.00–17.55) were significantly associated with the risk of LM. Finally, for individuals with a positive family history of hematologic malignancy (*n* cases = 709, *n* controls = 3376), the frequency of PVs was 5.4% in cases and 3.7% in controls (Table S5). PVs in *CHEK2* (OR = 2.70, 95% CI: 1.52–4.68) were significantly associated with increased risk of LM; *ATM* (OR = 2.27, 95% CI: 0.84–5.63) did not reach statistical significance, and *TP53* did not have enough mutation carriers to assess risk.

### Association of predisposition genes and lymphoid subtypes

**Fig. 2:**
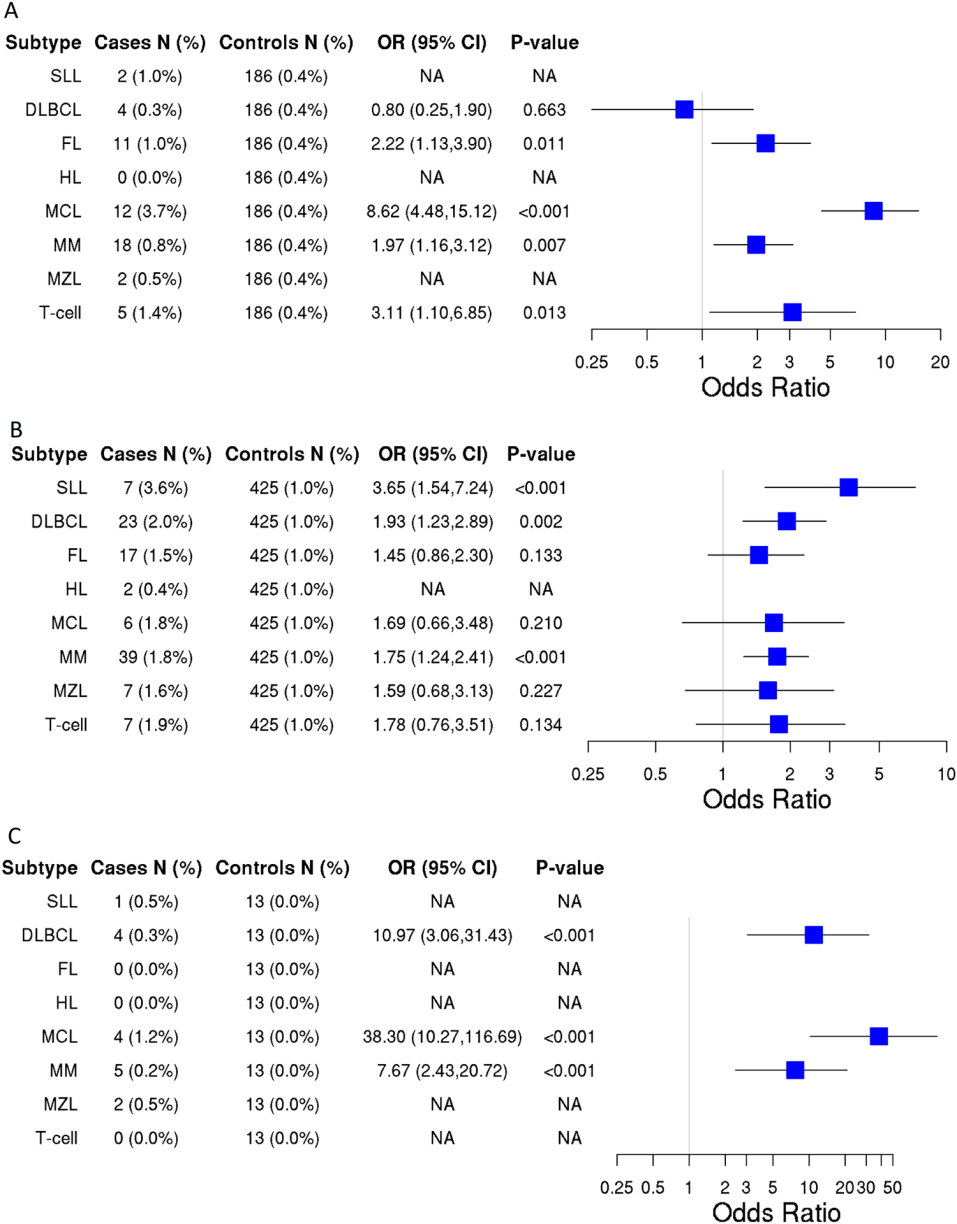
Risk of lymphoid malignancy subtype by cancer predisposition gene. Data shown for *ATM* (**A**)*, CHEK2* (**B**), and *TP53* (**C**). Gene selected based on being significantly associated with at least one lymphoid subtype. OR Odds Ratio, CI Confidence Interval. Estimates were adjusted for age and sex. NA denotes not applicable (too few events [<4] to calculate a stable odds ratio). SLL small lymphocytic lymphoma, DLBCL diffuse large B-cell lymphoma, FL follicular lymphoma, HL Hodgkin’s lymphoma, MCL mantle cell lymphoma, MM multiple myeloma, MZL marginal zone lymphoma.

Given the heterogenous nature of lymphoid malignancies, we further investigated the prevalence of PV in the cancer predisposition genes and risk associated with each subtype (Tables S6–S14). The collective frequency of PVs across the subtypes in the Mayo study ranged from 2.0% to 10.5%, with HL having the lowest frequency and MCL having the highest. PVs in *ATM, CHEK2, NBN*, and *TP53* were associated with at least one LM subtype, while all other genes showed no evidence of subtype specific associations (Fig. 2). PVs in *ATM* were associated with increased risk of FL (OR = 2.22, 95% CI: 1.13–3.90), MCL (OR = 8.62, 95% CI: 4.48–15.12), MM (OR = 1.97, 95% CI: 1.16–3.12), and T-cell (OR = 3.11, 95% CI: 1.16–6.85). PVs in *CHEK2* was associated with increased risk of SLL (OR = 3.65, 95% CI: 1.54–7.24), DLBCL (OR = 1.93, 95% CI: 1.23–2.89), and MM (OR = 1.75, 95% CI: 1.24–2.41). Additionally, OR estimates for FL, MCL, MZL, and T-cell were all elevated (OR~1.5 or higher) for *CHEK2*, but none were statistically significant. Finally, PVs in *TP53* were associated with increased risk of DLBCL (OR = 10.97, 95% CI: 3.06–31.43), MCL (OR = 38.30, 95% CI: 10.27–116.69), MM (OR = 7.67, 95% CI: 2.43–20.72), and other B-cell (OR = 16.12, 95% CI: 4.47–46.62).

### Patient characteristics of cases with PV

Given that *ATM*, *CHEK2*, and *TP53* were significantly associated with LM risk, we next investigated patient characteristics of cases with or without PV in these genes (Table 2). A total of 202 (6.8%) cases had a PV. Overall, cases who were PV carriers across these three genes had an elevated frequency of positive family history of hematologic malignancies compared to non-carrier cases (20.6% vs. 14.7%, *p* = 0.06), which was largely driven by the positive family history frequency of PV in *CHEK2* (22.7%). However, cases who were PV carriers vs. non-carriers had similar median age at diagnosis (median age 63), similar proportion of cases under age 60 (40.1% vs 41.1%, respectively, *p* = 0.78) and similar proportion of males (58.6% vs. 57.9%, respectively, *p* = 0.86). Lastly, 19.6% of cases with a PV had a prior history of solid tumor cancer and were not significantly different than the 15.6% of cases who were non-carriers (*p* = 0.27).

**Table 2:** Patient characteristics by pathogenic variant status overall and by gene.

	No Mutation (*N* = 6788)	Any Mutation (*N* = 202)	*ATM* (*N* = 57)	*CHEK2* (*N* = 125)	*TP53* (*N* = 20)
Age					
*N*	6660	202	57	125	20
Median	63	63	64	62	61
Range	18.0–94.0	20.0–90.0	25.0–90.0	20.0–89.0	37.0–79.0
Age category, *N* (%)					
Missing	4	0	0	0	0
<60	2721 (40.1%)	83 (41.1%)	20 (35.1%)	54 (43.2%)	9 (45.0%)
60+	4063 (59.9%)	119 (58.9%)	37 (64.9%)	71 (56.8%)	11 (55.0%)
Sex, *N* (%)					
Missing	3	0	0	0	0
Female	2812 (41.4%)	85 (42.1%)	20 (35.1%)	59 (47.2%)	6 (30.0%)
Male	3973 (58.6%)	117 (57.9%)	37 (64.9%)	66 (52.8%)	14 (70.0%)
Family history of hematologic malignancy, *N* (%)					
Missing	2162	66	19	37	10
No	3945 (85.3%)	108 (79.4%)	31 (81.6%)	68 (77.3%)	9 (90.0%)
Yes	681 (14.7%)	28 (20.6%)	7 (18.4%)	20 (22.7%)	1 (10.0%)
Personal history of prior solid tumor cancer, *N* (%)					
Missing	3376	100	27	63	10
No	2881 (84.4%)	82 (80.4%)	27 (90.0%)	48 (77.4%)	7 (70.0%)
Yes	531 (15.6%)	20 (19.6%)	3 (10.0%)	14 (22.6%)	3 (30.0%)

Family history of hematologic malignancy is self report.

Personal history of prior solid tumor cancer is self report.

## Discussion

In this case-control study of 6990 cases and 42,632 controls, we report the prevalence of PV in 19 cancer predisposition genes commonly found on genetic testing panels and provide estimates of the risk of LM associated with these PV. Overall, cases had a significantly higher prevalence of PV compared to controls. *ATM* and *CHEK2* were significantly associated with an approximately 2-fold increased risk, while *TP53* was associated with a 9-fold increased risk of LM. These associations were validated using 4772 LM cases and 389,731 controls from the UKB, further confirming that these cancer predisposition genes are associated with LM. Furthermore, PV in *ATM, CHEK2*, and *TP53* were associated with moderate to high risk of specific LM subtypes, but not all subtypes, highlighting the heterogeneity of LM.

The cancer predisposition genes investigated here are associated with the risk of an array of solid tumor cancers, with some evidence in LM. In an analysis of 23 hereditary cancer genes from 3 cohorts, carriers of PV in *ATM* had a 1.9-fold increased risk of NHL [33]. *CHEK2* was associated with 2-fold elevated risk of NHL in a Polish study [34] and was replicated in a Czech study (2.9-fold increase) [35]. Lymphoma risk was associated with PV in *ATM* (2.6-fold increase) and *TP53* (5.2-fold increase) in a recent study using a Japanese biobank [26]. Our results for *ATM*, *CHEK2*, and *TP53* from both the Mayo Clinic and UKB cohorts are consistent with these smaller studies. Collectively, these results provide strong evidence that these cancer predisposition genes are associated with LM. Conversely, a few studies have suggested that the *BRCA* genes are associated with increased risk of NHL, HL, or MM including *BRCA1* (7.7-fold increase) and *BRCA2* (5.9-fold increase) in the Japanese population [26] and *BRCA2* (3.3-fold increase) in pediatric or adolescent lymphoma survivors from St. Jude Life cohort [25], and *BRCA1* (3.9-fold increase) and *BRCA2* (7.0-fold increase) in MM cases compared to gnomAD controls [36]. We did not find evidence of an association between PV in either gene with the risk of LM. In support of our *BRCA1* and *BRCA2* results, there was not an increased frequency of PV in LM cases compared to controls in the UKB for *BRCA1* (0.2% in cases vs. 0.1% in controls) or *BRCA2* (0.3% in cases vs. 0.3% in controls). Further research is required to understand these discrepancies, including the need for replication in these target populations as well as the possibility of population specific associations through founder mutations, although this is unlikely given the similar frequencies of PV in the control population in the Japanese population and our study; an age related association that could not be detected in our study due to older age of onset compared to the St Jude study; or comparison to gnomAD controls which may have a different population structure compared to ascertainment of cases.

As with the heterogenous nature of LM, we also observed heterogeneity with regard to genes associated with LM. The 3 genes (*ATM, CHEK2, TP53*) associated with overall LM in our study were generally associated with multiple subtypes but not consistently the same subtypes. For example, the risk of DLBCL was associated with PV in *CHEK2* and *TP53* but not *ATM*. FL was associated with PV in *ATM* but not *CHEK2* nor *TP53*. Studies investigating the relationship between PV in cancer predisposition genes in MM are lacking. Here, MM was the only subtype associated with PV in all three genes, *ATM, CHEK2*, and *TP53*. In the Japanese population, PV collectively in *ATM*, *BRCA1*, *BRCA2*, and *TP53* was most highly associated with the risk of MCL [26]. Both *ATM* and *TP53* were highly associated with the risk of MCL in our study, with 8.62- and 38.3-fold increased risk, respectively. HL had too few cases with a PV in any gene to estimate risk, which may be suggestive that genes predominately associated with the DNA damage response are not associated with HL. It is worth noting that *CHEK2* was consistently elevated (~2-fold) for risk of each subtype (except for HL) but was not always statistically significant.

All three genes found to be associated with LM in this study are directly related to the DNA damage response (DDR) pathway. Mutations in either *ATM* or *CHEK2* disrupt the repair mechanisms for double-strand breaks, impairing the cell’s ability to repair these breaks [37, 38]. Mouse studies have shown that *ATM* or *CHEK2* deficient mice often develop lymphoma [39–41]. *TP53* is important to cell cycle checkpoints. The absence of functional *TP53* results in checkpoint failures and unrepaired double-strand breaks. As with *ATM* and *CHEK2*, mutations in *TP53* predispose mice to lymphoma [42, 43]. Our large study confirms prior mouse models that the association between these genes, which are integral to DNA repair, are associated with risk LM.

The presence of PV in cancer predispositions genes has often been shown to be associated with patient and clinical characteristics, such as younger age of onset of disease, family history, or histology[14, 44]. In this study, we found no evidence of an association between PV carriers and non-carriers with age of onset, sex, or enrichment of family history of hematological malignancies in our cases. These results are consistent with those reported in a study from Japan [26]. Our null results with respect to patient characteristics at the time of diagnosis provide little information as to which individuals carry a PV in one of these three genes. Therefore, these results do not inform NCCN criteria to identify individuals that might benefit from genetic testing based on risk of LM, nor do they support the need for routine screening given the moderate effect sizes of the associations. However, this does not mean these results have no clinical utility. Individuals who do qualify for genetic testing based on NCCN criteria for other malignancies can be informed of their risk of LM. Further, family members that may carry a PV in a cancer predisposition gene may benefit from understanding their risks of LM. Additional research is required to investigate the clinical characteristics, response to therapy, toxicity, and survival of patients with LM that carry these inherited PV. This information may inform clinical management, alternative therapies, and surveillance. Finally, the WHO-HEAM5 Classification acknowledges that with the growing number of lymphomas linked to germline predisposition genes, there is a need to incorporate these findings into the classification (as for other organ sites) [2]. This is currently recommended as using conventional criteria for the diagnosis with a notation for any germline associations. It is not yet clear how many germline associations may yet be identified.

This study is not without limitations. This study population is almost exclusively of European ancestry and may not completely generalize to non-European ancestries and should be a focus of future studies. As our DNA source was peripheral blood, some mutations could be somatic (i.e. clonal hematopoiesis of indeterminate potential, or CHIP), most prominently *TP53* [45, 46]. Although we did not confirm our mutations as inherited or somatic through orthogonal sequencing, our variant allele fractions were normally distributed around 0.5 (Fig. S1), fractions that are in line with inherited mutations. To mitigate including CHIP variants, we restricted the VAF to be within 0.3–0.7 for *TP53;* however, we cannot rule out that a small percentage of *TP53* mutations included in this study were not large clones. Nonetheless, our findings agree with other studies that have investigated inherited PV in *TP53* LM patients [26, 47].

In summary, we identified that established cancer predisposition genes are associated with a moderate to high risk of LM overall and with LM subtypes. These results can be used to better inform individuals of their risk of LM. Furthermore, this study demonstrates that rare inherited genetics is an additional source of variation that expands our understanding of the genetic architecture of LM risk.

## Supplementary information

Supplementary Tables

Supplementary Figure 1

## Data Availability

The data that support the findings of this study are available from the corresponding author upon reasonable request. Variant level summary data may be found in a data supplement available with the online version of this article.

## References

[1] Teras LR, DeSantis CE, Cerhan JR, Morton LM, Jemal A, Flowers CR. 2016 US lymphoid malignancy statistics by World Health Organization subtypes. CA Cancer J Clin. 2016;66:443–59. 27618563 10.3322/caac.21357

[2] Alaggio R, Amador C, Anagnostopoulos I, Attygalle AD, Araujo IBO, Berti E, et al. The 5th edition of the World Health Organization Classification of Haematolymphoid Tumours: Lymphoid Neoplasms. Leukemia. 2022;36:1720–48. 35732829 10.1038/s41375-022-01620-2 PMC9214472

[3] Siegel RL, Miller KD, Wagle NS, Jemal A. Cancer statistics, 2023. CA Cancer J Clin. 2023;73:17–48. 36633525 10.3322/caac.21763

[4] Thun MJ, Linet MS, Cerhan JR, Haiman C, Schottenfeld D Schottenfeld and Fraumeni cancer epidemiology and prevention. Fourth edition. ed. New York, NY: Oxford University Press; 2018. xix, 1308 pages p.

[5] Cerhan JR, Slager SL. Familial predisposition and genetic risk factors for lymphoma. Blood. 2015;126:2265–73. 26405224 10.1182/blood-2015-04-537498 PMC4643002

[6] Goldin LR, Bjorkholm M, Kristinsson SY, Turesson I, Landgren O. Highly increased familial risks for specific lymphoma subtypes. Br J Haematol. 2009;146:91–4. 19438470 10.1111/j.1365-2141.2009.07721.x PMC2702464

[7] Sud A, Chattopadhyay S, Thomsen H, Sundquist K, Sundquist J, Houlston RS, et al. Analysis of 153 115 patients with hematological malignancies refines the spectrum of familial risk. Blood. 2019;134:960–9. 31395603 10.1182/blood.2019001362 PMC6789511

[8] Cerhan JR, Berndt SI, Vijai J, Ghesquieres H, McKay J, Wang SS, et al. Genome-wide association study identifies multiple susceptibility loci for diffuse large B cell lymphoma. Nat Genet. 2014;46:1233–8. 25261932 10.1038/ng.3105 PMC4213349

[9] Kleinstern G, Yan H, Hildebrandt MAT, Vijai J, Berndt SI, Ghesquieres H, et al. Inherited variants at 3q13.33 and 3p24.1 are associated with risk of diffuse large B-cell lymphoma and implicate immune pathways. Hum Mol Genet. 2020;29:70–9. 31600786 10.1093/hmg/ddz228 PMC7001601

[10] Law PJ, Berndt SI, Speedy HE, Camp NJ, Sava GP, Skibola CF, et al. Genome-wide association analysis implicates dysregulation of immunity genes in chronic lymphocytic leukaemia. Nat Commun. 2017;8:14175. 28165464 10.1038/ncomms14175 PMC5303820

[11] Skibola CF, Berndt SI, Vijai J, Conde L, Wang Z, Yeager M, et al. Genome-wide association study identifies five susceptibility loci for follicular lymphoma outside the HLA region. Am J Hum Genet. 2014;95:462–71. 25279986 10.1016/j.ajhg.2014.09.004 PMC4185120

[12] Went M, Sud A, Forsti A, Halvarsson BM, Weinhold N, Kimber S, et al. Author Correction: Identification of multiple risk loci and regulatory mechanisms influencing susceptibility to multiple myeloma. Nat Commun. 2019;10:213. 30631080 10.1038/s41467-018-08107-8 PMC6328616

[13] Breast Cancer Association C, Dorling L, Carvalho S, Allen J, Gonzalez-Neira A, Luccarini C, et al. Breast cancer risk genes - association analysis in more than 113,000 women. N Engl J Med. 2021;384:428–39. 33471991 10.1056/NEJMoa1913948 PMC7611105

[14] Hu C, Hart SN, Gnanaolivu R, Huang H, Lee KY, Na J, et al. A population-based study of genes previously implicated in breast cancer. N Engl J Med. 2021;384:440–51. 33471974 10.1056/NEJMoa2005936 PMC8127622

[15] Kurian AW, Hughes E, Handorf EA, Gutin A, Allen B, Hartman AR, et al. Breast and ovarian cancer penetrance estimates derived from germline multiple-gene sequencing results in women. JCO Precis Oncol. 2017;1:1–12. 10.1200/PO.16.00066 35172496

[16] Seppala EH, Ikonen T, Mononen N, Autio V, Rokman A, Matikainen MP, et al. CHEK2 variants associate with hereditary prostate cancer. Br J Cancer. 2003;89:1966–70. 14612911 10.1038/sj.bjc.6601425 PMC2394451

[17] Yadav S, Hart SN, Hu C, Hillman D, Lee KY, Gnanaolivu R, et al. Contribution of inherited DNA-repair gene mutations to hormone-sensitive and castrate-resistant metastatic prostate cancer and implications for clinical outcome. JCO Precis Oncol. 2019;3. 10.1200/PO.19.00067 PMC7446380 32923857

[18] Siolek M, Cybulski C, Gasior-Perczak D, Kowalik A, Kozak-Klonowska B, Kowalska A, et al. CHEK2 mutations and the risk of papillary thyroid cancer. Int J Cancer. 2015;137:548–52. 25583358 10.1002/ijc.29426

[19] Bychkovsky BL, Agaoglu NB, Horton C, Zhou J, Yussuf A, Hemyari P, et al. Differences in cancer phenotypes among frequent CHEK2 variants and implications for clinical care-checking CHEK2. JAMA Oncol. 2022;8:1598–606. 36136322 10.1001/jamaoncol.2022.4071 PMC9501803

[20] Hall MJ, Bernhisel R, Hughes E, Larson K, Rosenthal ET, Singh NA, et al. Germline pathogenic variants in the Ataxia Telangiectasia Mutated (ATM) gene are associated with high and moderate risks for multiple cancers. Cancer Prev Res. 2021;14:433–40. 10.1158/1940-6207.CAPR-20-0448 PMC8026745 33509806

[21] Daly MB, Pal T, Maxwell KN, Churpek J, Kohlmann W, AlHilli Z, et al. NCCN guidelines(R) insights: genetic/familial high-risk assessment: breast, ovarian, and pancreatic, version 2.2024. J Natl Compr Cancer Netw. 2023;21:1000–10. 10.6004/jnccn.2023.0051 37856201

[22] McBride KA, Ballinger ML, Killick E, Kirk J, Tattersall MH, Eeles RA, et al. Li-Fraumeni syndrome: cancer risk assessment and clinical management. Nat Rev Clin Oncol. 2014;11:260–71. 24642672 10.1038/nrclinonc.2014.41

[23] Bansidhar BJ. Extracolonic manifestations of lynch syndrome. Clin Colon Rectal Surg. 2012;25:103–10. 23730225 10.1055/s-0032-1313781 PMC3423888

[24] Ripperger T, Schlegelberger B. Acute lymphoblastic leukemia and lymphoma in the context of constitutional mismatch repair deficiency syndrome. Eur J Med Genet. 2016;59:133–42. 26743104 10.1016/j.ejmg.2015.12.014

[25] Wang Z, Wilson CL, Armstrong GT, Hudson MM, Zhang J, Nichols KE, et al. Association of germline BRCA2 mutations with the risk of pediatric or adolescent non-Hodgkin lymphoma. JAMA Oncol. 2019;5:1362–4. 31343663 10.1001/jamaoncol.2019.2203 PMC6659356

[26] Usui Y, Iwasaki Y, Matsuo K, Endo M, Kamatani Y, Hirata M, et al. Association between germline pathogenic variants in cancer-predisposing genes and lymphoma risk. Cancer Sci. 2022;113:3972–9. 36065483 10.1111/cas.15522 PMC9633290

[27] Cerhan JR, Link BK, Habermann TM, Maurer MJ, Feldman AL, Syrbu SI, et al. Cohort profile: the lymphoma Specialized Program of Research Excellence (SPORE) Molecular Epidemiology Resource (MER) Cohort Study. Int J Epidemiol. 2017;46:1753–4i. 29025017 10.1093/ije/dyx119 PMC5837578

[28] Swerdlow SH, Campo E, Harris NL, Jaffe ES, Pileri S, Stein H, et al. WHO classification of tumours of haematopoietic and lymphoid tissues. Lyon: International Agency for Research on Cancer; 2017.

[29] Olson JE, Ryu E, Hathcock MA, Gupta R, Bublitz JT, Takahashi PY, et al. Characteristics and utilisation of the Mayo Clinic Biobank, a clinic-based prospective collection in the USA: cohort profile. BMJ Open. 2019;9. 10.1136/bmjopen-2019-032707 PMC6858142 31699749

[30] Kocher JP, Quest DJ, Duffy P, Meiners MA, Moore RM, Rider D, et al. The Biological Reference Repository (BioR): a rapid and flexible system for genomics annotation. Bioinformatics. 2014;30:1920–2. 24618464 10.1093/bioinformatics/btu137 PMC4071205

[31] Weitzel JN, Chao EC, Nehoray B, Van Tongeren LR, LaDuca H, Blazer KR, et al. Somatic TP53 variants frequently confound germ-line testing results. Genet Med. 2018;20:809–16. 29189820 10.1038/gim.2017.196 PMC5976505

[32] Sudlow C, Gallacher J, Allen N, Beral V, Burton P, Danesh J, et al. UK biobank: an open access resource for identifying the causes of a wide range of complex diseases of middle and old age. PLoS Med. 2015;12:e1001779. 25826379 10.1371/journal.pmed.1001779 PMC4380465

[33] Zeng C, Bastarache LA, Tao R, Venner E, Hebbring S, Andujar JD, et al. Association of pathogenic variants in hereditary cancer genes with multiple diseases. JAMA Oncol. 2022;8:835–44. 35446370 10.1001/jamaoncol.2022.0373 PMC9026237

[34] Cybulski C, Gorski B, Huzarski T, Masojc B, Mierzejewski M, Debniak T, et al. CHEK2 is a multiorgan cancer susceptibility gene. Am J Hum Genet. 2004;75:1131–5. 15492928 10.1086/426403 PMC1182149

[35] Havranek O, Kleiblova P, Hojny J, Lhota F, Soucek P, Trneny M, et al. Association of germline CHEK2 gene variants with risk and prognosis of non-Hodgkin lymphoma. PLoS ONE. 2015;10:e0140819. 26506619 10.1371/journal.pone.0140819 PMC4624763

[36] Thibaud S, Subaran RL, Newman S, Lagana A, Melnekoff DT, Bodnar S, et al. Multiple myeloma risk and outcomes are associated with pathogenic germline variants in DNA repair genes. Blood Cancer Discov. 2024;5:428–41. 39283238 10.1158/2643-3230.BCD-23-0208 PMC11528192

[37] Bartek J, Lukas J. Chk1 and Chk2 kinases in checkpoint control and cancer. Cancer Cell. 2003;3:421–9. 12781359 10.1016/s1535-6108(03)00110-7

[38] Lavin MF, Kozlov S. ATM activation and DNA damage response. Cell Cycle. 2007;6:931–42. 17457059 10.4161/cc.6.8.4180

[39] Bahassi el M, Robbins SB, Yin M, Boivin GP, Kuiper R, van Steeg H, et al. Mice with the CHEK2*1100delC SNP are predisposed to cancer with a strong gender bias. Proc Natl Acad Sci USA. 2009;106:17111–6. 19805189 10.1073/pnas.0909237106 PMC2761365

[40] Hathcock KS, Padilla-Nash HM, Camps J, Shin DM, Triner D, Shaffer AL 3rd, et al. ATM deficiency promotes development of murine B-cell lymphomas that resemble diffuse large B-cell lymphoma in humans. Blood. 2015;126:2291–301. 26400962 10.1182/blood-2015-06-654749 PMC4643004

[41] Tepsuporn S, Hu J, Gostissa M, Alt FW. Mechanisms that can promote peripheral B-cell lymphoma in ATM-deficient mice. Cancer Immunol Res. 2014;2:857–66. 24913718 10.1158/2326-6066.CIR-14-0090 PMC4156541

[42] Gostissa M, Bianco JM, Malkin DJ, Kutok JL, Rodig SJ, Morse HC 3rd, et al. Conditional inactivation of p53 in mature B cells promotes generation of nongerminal center-derived B-cell lymphomas. Proc Natl Acad Sci USA. 2013;110:2934–9. 23382223 10.1073/pnas.1222570110 PMC3581964

[43] Rowh MA, DeMicco A, Horowitz JE, Yin B, Yang-Iott KS, Fusello AM, et al. Tp53 deletion in B lineage cells predisposes mice to lymphomas with oncogenic translocations. Oncogene. 2011;30:4757–64. 21625223 10.1038/onc.2011.191

[44] Mavaddat N, Barrowdale D, Andrulis IL, Domchek SM, Eccles D, Nevanlinna H, et al. Pathology of breast and ovarian cancers among BRCA1 and BRCA2 mutation carriers: results from the Consortium of Investigators of Modifiers of BRCA1/2 (CIMBA). Cancer Epidemiol Biomark Prev. 2012;21:134–47. 10.1158/1055-9965.EPI-11-0775 PMC3272407 22144499

[45] Genovese G, Kähler AK, Handsaker RE, Lindberg J, Rose SA, Bakhoum SF, et al. Clonal hematopoiesis and blood-cancer risk inferred from blood DNA sequence. N Engl J Med. 2014;371:2477–87. 25426838 10.1056/NEJMoa1409405 PMC4290021

[46] Jaiswal S, Fontanillas P, Flannick J, Manning A, Grauman PV, Mar BG, et al. Age-related clonal hematopoiesis associated with adverse outcomes. N Engl J Med. 2014;371:2488–98. 25426837 10.1056/NEJMoa1408617 PMC4306669

[47] Scott AJ, Tokaz MC, Jacobs MF, Chinnaiyan AM, Phillips TJ, Wilcox RA. Germline variants discovered in lymphoma patients undergoing tumor profiling: a case series. Fam Cancer. 2021;20:61–5. 32504211 10.1007/s10689-020-00192-3 PMC7719097

