# Supplementary material for: Risk of lymphoid malignancy associated with cancer predisposition genes
Source: Blood Cancer J. 2025 Apr 19;15(1):71. doi: 10.1038/s41408-025-01283-z (PMC12009404; doi:10.1038/s41408-025-01283-z)
Supplement: Supplementary file 2 — Supplementary Figure 1 [file 41408_2025_1283_MOESM2_ESM.docx]

**Supplemental Figure 1. Distribution of variant allele fraction of pathogenic variants by gene**
